# The CO-VID D-Lemma: A Call for Action

**DOI:** 10.3390/nu14050963

**Published:** 2022-02-24

**Authors:** Michael F. Holick

**Affiliations:** Section Endocrinology, Diabetes, Nutrition and Weight Management, Department of Medicine, Boston University Medical Center, Boston, MA 02118, USA; mfholick@bu.edu

It is remarkable how an invisible, inanimate particle—severe acute respiratory syndrome coronavirus 2 (SARS-CoV-2, COVID-19)—that is hell-bent on reproducing itself was able to bring our modern civilization to its knees. History, however, continues to repeat itself, even in the era of modern medicine and RNA vaccines. Although in the past two years there has been a plethora of publications documenting that vitamin D deficiency increases the risk for infectivity, morbidity and mortality, this documentation has fallen on deaf ears for most health care providers and healthcare regulators.

As early as 1300 BC Akenaten appreciated the life-giving properties of sun exposure. This was followed by Hippocrates prescribing heliotherapy for both medical and psychological purposes. Sniadecki, in 1822, and Palm, in 1890, made the connection between sun deprivation and the devastating bone disease rickets [1]. At the same time, Florence Nightingale, in the 1850s, identified sunlight as the key to promoting health in hospitals, and wrote: “Direct sunlight, not only daylight, is necessary for speedy recovery”. CJB Williams, in 1849, documented that cod liver oil was an effective treatment for tuberculosis (TB) [2]. In 1903, Neils Finsen received the Nobel Prize for his observation that concentrated light from an electric arc lamp, or from sunlight, was an effective treatment for the disfiguring skin disease caused by TB. In 1921, Hess and Unger reported a dramatic improvement in rickets in children exposed to sunlight on the roof of their New York City Hospital. The connection between the antirachitic properties of ultraviolet radiation and vitamin D containing cod liver oil was made when rachitic rodents that were exposed to cod liver oil or ultraviolet radiation received the same skeletal benefit [1].

At the same time that sunlight exposure was found to have antirachitic and antituberculosis properties, it was reported that Spanish flu patients in 1918, who were treated with direct sunlight in the outside hospital at Camp Brooks in Massachusetts, fared better than those not exposed to direct sunlight [3]. An analysis of latitude and solar ultraviolet irradiation with Spanish flu fatalities revealed that living in northern latitudes, compared to southern latitudes, in the United States increased the risk of mortality by more than 50% [4]. This is also in line with what Hope Simpson observed. He reported that there was a seasonal stimulus for the flu season, which reaches its peak incidence in northern and southern latitudes in the winter at a time when little, if any, vitamin D is produced from sun exposure [1,5].

The sunshine vitamin D, either obtained naturally from direct sun exposure on the skin or from the diet, is metabolized in the liver into 25-hydroxyvitamin D [25(OH)D, the major circulating form measured to determine vitamin D status]. 25(OH)D is then transformed in the kidneys to its active form, 1,25-dihydroxyvitamin D [1,25(OH)2D], which is responsible for regulating calcium and bone metabolism by interacting with its vitamin D nuclear receptor (VDR) in the intestine and bone [6].

Most cells have a VDR, and many have the capacity to convert 25(OH)D to 1,25(OH)2D, including immune cells. Only when they are activated do B and T lymphocytes express a VDR. Macrophages, activated monocytes and dendritic cells produce 1,25(OH)2D to regulate their function, including producing cathelicidin, a natural virucidal and bactericidal peptide that is responsible for innate host defense against microbial pathogens [6,7,8]. In addition, these cells produce 1,25(OH)2D which is released locally to interact with the VDR in activated T and B lymphocytes and respiratory epithelial cells, to help regulate adaptive immune responses for combating microbial pathogens. The locally produced 1,25(OH)2D can also play a role in reducing the ability of the SARS-CoV-19 to enter the respiratory epithelial cells. It does this by suppressing the expression of membrane-bound angiotensin converting enzyme 2 (ACE2), which is the receptor for the spike protein on the SARS-CoV-2. In addition, COVID-19 infection may downregulate ACE2, which in turn, could lead to excessive accumulation of angiotensin II. High levels of angiotensin II may cause acute respiratory distress syndrome (ARDS), myocarditis and cardiac injury [9,10]. Renin, on the other hand, is a proteolytic enzyme and a positive regulator of angiotensin II. 1,25(OH)2D3 was shown in an animal model to prevent angiotensin II accumulation and to decrease the proinflammatory activity of angiotensin II by suppressing the release of renin. This could be beneficial in patients infected with COVID-19 by reducing the risk of ARDS, myocarditis and cardiac injury [10].

In the pre-COVID-19 era, vitamin D supplementation was shown to reduce the risk of upper respiratory tract infections. A meta-analysis of 25 randomized controlled studies showed that vitamin D supplementation can protect against the development of acute respiratory tract infections, compared with a placebo (odds ratio 0.88; 95% CI: 0.81–0.96) [11]. Additionally, vitamin D has been proposed to be an adjunctive treatment for reducing morbidity and mortality resulting from sepsis and critical illness, although randomized clinical trials have yielded mixed results [12].

There continues to be a heated debate as to whether improvement in vitamin D status has any benefit in reducing infectivity, morbidity and mortality associated with COVID-19. There have been several observational and association studies reporting that a higher vitamin D status is associated with a significant reduction in being infected with this deadly virus. A study was conducted on more than 190,000 COVID-19-positive patients, that included individuals of all ethnicities, within an age range of 40–64 years, and from all 50 states in the United States. After taking into account a multivariable logistic model that adjusted for all of the included demographic factors, including the season, latitudes, both sexes, races/ethnicities and age ranges, it was concluded that SARS-CoV-2 positivity was strongly and inversely associated with serum concentrations of 25(OH)D. For the entire population, those who had a circulating concentration of 25(OH)D < 50 nmol/L (20 ng/mL) had a 54% higher positivity rate compared to those who had a blood concentration of 75–85 nmol/L (30–34 ng/mL). The risk of infection continued to decline until serum concentrations reached 137.5 nmol/l (55 ng/mL) [13]. This observation is consistent with previously published observations reporting an inverse relationship between risk for respiratory viral pathogens, including influenza, and serum 25(OH)D concentrations [14]. Several much smaller studies in Chicago, Spain, Korea, the United Kingdom and Israel have all reported that vitamin D deficiency was a risk factor for COVID-19 infectivity [8,14]. What is also important to recognize from these studies is that the determination for infectivity was selected from individuals who had a serum 25(OH)D that was taken within 12 months prior to being tested for COVID-19. This means that it is likely that their blood levels were relatively the same for that entire period of time. This could help to explain the disparate observations where some studies suggest that patients who are vitamin D sufficient have less morbidity and mortality, whereas intervention studies with pharmacologic dose(s) of vitamin D were not found to have any benefit when compared to a placebo [8,15]. Seal et al. [16] reported on 4599 veterans who had a positive SARS-CoV-2 test. Nine hundred sixty-four of the veterans were hospitalized with COVID-19. After adjusting for all covariates, including race/ethnicity and poverty, there was a significant independent inverse dose–response relationship between increasing the serum concentrations of 25(OH)D from 37.5–150 nmol/L (15–60 ng/mL) and a decrease in hospitalization by 23%, and of mortality by 48%. These results confirm several other observational studies including a smaller Iranian study that reported that hospitalized patients, of which 74% had severe COVID-19, had a decreased risk for the severity of clinical outcomes including unconsciousness and hypoxia, and a 51.5% reduced risk of mortality for those 40 years and older [17]. These observations were related to significant reductions in C-reactive protein and an increased lymphocyte percentage.

These studies provide compelling evidence that being vitamin D sufficient, defined by the Endocrine Society [18] as having a 25(OH)D level of at least 75 nmol/L (30 ng/mL), substantially reduced infectivity, morbidity and mortality from this highly infectious virus. Most of these studies also suggested that increased continuous 25(OH)D concentrations of up to 150 nmol/L (60 ng/mL) continue to provide additional reduction in these clinical parameters [8,16,17].

The fact that a few randomized controlled trials that gave extremely high dose(s) of vitamin D showed little benefit is not at all unexpected. Giving a high dose(s) of vitamin D to a vitamin D deficient patient is like placing a Band-Aid on an arterial bleeding wound. It takes time for (a) vitamin D to be converted to 25(OH)D; (b) this metabolite to be converted in immune cells to 1,25(OH)2D; and (c) 1,25(OH)2D to interact with its VDR to enhance innate and adaptive immune responses. During this time, the virus is rapidly multiplying and can overwhelm the immune response. There is, however, one potential option to help speed up the process of vitamin D carrying out its innate and adaptive immune responses. Patients with COVID-19 entering the hospital could receive 25(OH)D3 (calcifediol) to rapidly improve their vitamin D status, and thereby quickly provide the immune system with its immunologic regulating precursor; it can then be rapidly converted to 1,25(OH)2D. A Spanish study evaluated the potential benefit of giving calcifediol to COVID-19 patients at the time of hospitalization. The patients all received standard care, and a randomized 2:1 ratio of calcifediol to standard care alone. They reported that only 2% of the patients receiving calcifediol required intensive care unit (ICU) admission, whereas 50% of those on standard care were admitted to the ICU [19]. A double-blind placebo-controlled trial in Iran reported that in 106 hospitalized patients who had a serum concentration of 25(OH)D < 75 nmol/L (<30 ng/mL), those who received calcifediol had a lower trend for hospitalization, ICU duration, need for ventilator assistance and mortality, compared to the placebo group. They also observed that the improved outcomes in calcifediol treated patients were associated with a higher percentage of lymphocyte count and a lower neutrophil–lymphocyte ratio, which is a surrogate marker for decreased inflammatory activity [20].

In its totality, to date, the majority of studies evaluating vitamin D status suggest that being vitamin D sufficient in the era of COVID-19 reduces the risk of infection and clinical complications associated with this infection. A rapid improvement in vitamin D status at the time of hospitalization with calcifediol may also help to reduce the length of stay, ICU admissions and mortality in COVID-19 patients.

To put this into perspective, it was reported that a five-day treatment with molnupiravir, which was hailed as a huge advance in the treatment of COVID-19, reduced hospitalizations by 50%, and at a significant cost. On the other hand, maintaining vitamin D sufficiency with vitamin D supplementation, at relatively little cost, reduces the risk of being infected by more than 50%, thereby reducing hospitalizations caused by this infection. For COVID-19 patients entering the hospital, if they are vitamin D sufficient, they are more likely to experience shorter hospitalization and no ICU admission, and are more likely to leave the hospital.

It is reasonable to follow the Endocrine Society Practice Guidelines on Vitamin D to maintain vitamin D sufficiency. The Guidelines recommend 400–1000 IUs, 600–1000 IUs and 1500–2000 IUs for infants up to 1 year, children up to 18 years and all adults, respectively. Due to the dilution effect of body fat, the guidelines recommend that adults with a BMI > 30 require 2–3 times more vitamin D to be vitamin D sufficient [18]. These recommendations are reasonable, and will achieve a blood concentration of 25(OH)D in the range of 75–100 nmol/L (30–40 ng/mL). To achieve higher levels requires additional vitamin D supplementation. Every 100 IUs ingested will raise the blood concentration of 25(OH)D by approximately 1.5–2.5 nmol/L (0.6–1.0 nl/mL) [18]. Therefore, to achieve blood levels in the preferred range as recommended by the Endocrine Society would require ingesting 4000–6000 IUs daily for adults. It is wise in this COVID-19 pandemic to have sensible sun exposure, as was recommended more than 100 years ago during the Spanish flu pandemic, and to maintain a healthy vitamin D status with dietary and supplemental vitamin D.

## Data Availability

Not applicable.
